# Lost productivity due to premature mortality in developed and emerging countries: an application to smoking cessation

**DOI:** 10.1186/1471-2288-12-87

**Published:** 2012-06-25

**Authors:** Joseph Menzin, Jeno P Marton, Jordan A Menzin, Richard J Willke, Rebecca M Woodward, Victoria Federico

**Affiliations:** 1 Boston Health Economics, Inc, 20 Fox Road, Waltham, MA 02451, USA; 2 Pfizer, Inc, 235 East 42nd Street, New York, NY 10017, USA

## Abstract

**Background:**

Researchers and policy makers have determined that accounting for productivity costs, or “indirect costs,” may be as important as including direct medical expenditures when evaluating the societal value of health interventions. These costs are also important when estimating the global burden of disease. The estimation of indirect costs is commonly done on a country-specific basis. However, there are few studies that evaluate indirect costs across countries using a consistent methodology.

**Methods:**

Using the human capital approach, we developed a model that estimates productivity costs as the present value of lifetime earnings (PVLE) lost due to premature mortality. Applying this methodology, the model estimates productivity costs for 29 selected countries, both developed and emerging. We also provide an illustration of how the inclusion of productivity costs contributes to an analysis of the societal burden of smoking. A sensitivity analysis is undertaken to assess productivity costs on the basis of the friction cost approach.

**Results:**

PVLE estimates were higher for certain subpopulations, such as men, younger people, and people in developed countries. In the case study, productivity cost estimates from our model showed that productivity loss was a substantial share of the total cost burden of premature mortality due to smoking, accounting for over 75 % of total lifetime costs in the United States and 67 % of total lifetime costs in Brazil. Productivity costs were much lower using the friction cost approach among those of working age.

**Conclusions:**

Our PVLE model is a novel tool allowing researchers to incorporate the value of lost productivity due to premature mortality into economic analyses of treatments for diseases or health interventions. We provide PVLE estimates for a number of emerging and developed countries. Including productivity costs in a health economics study allows for a more comprehensive analysis, and, as demonstrated by our illustration, can have important effects on the results and conclusions.

## Background

In the current climate of rising health care costs, evaluations demonstrating the economic value of new health care interventions are increasingly becoming a necessity [1-4]. These economic evaluations include assessment of the “costs” and “benefits” of the health care intervention under consideration. Costs typically include direct costs such as medical expenditures as well as indirect costs due to lost productivity from morbidity or premature mortality [2,3,5]. Similarly, burden of disease studies, which help motivate interest and investment in new health care interventions, usually require assessment of both direct and indirect costs.

There is a general consensus that, when feasible, researchers should conduct their health economic analyses from the societal perspective, as it is the most comprehensive [2-7]. Moreover, for an analysis to truly be from the societal point of view, productivity costs must be taken into account [2,3,6]. For example, a disease may affect society not only through the financial transactions related to the exchange of goods and services to treat that disease, but also through the loss of individuals’ contributions to society over their lifetimes due to having that disease. There is disagreement on whether to include productivity costs when cost-utility analyses are undertaken [3-5,8]. However, as many as one-third of published cost-utility studies have incorporated indirect costs [2], and other forms of economic evaluation (e.g., cost-benefit analysis) call for the inclusion of such costs on a routine basis [5].

In the health economics literature, there are two competing methodologies for estimating productivity costs: the human capital approach and the friction cost method [9-12]. The human capital approach assumes no unemployment and captures all lost productivity due to disease mortality by assuming individuals who died prematurely would have worked full time until the end of their working lives. In contrast, the friction cost method captures lost productivity costs only until a worker would likely be replaced by someone currently unemployed plus the transaction costs associated with identifying the replacement worker [13]. The human capital approach can also be adapted to include unpaid labor, such as household work, in productivity cost estimates [3] and has been more widely accepted and recommended [6,14-16].

This paper describes a model that we developed to estimate productivity costs due to premature mortality for a range of developed and emerging countries around the world. We elected to use the human capital approach, but we included sensitivity analyses that employed the friction cost method. Utilizing a variety of inputs, including population demographics, the percentage of working age people employed or seeking employment (i.e., the labor force participation rate), and life expectancy by age and gender, we apply a consistent methodology across countries. To our knowledge, there are no existing models that provide estimates of productivity costs for such a wide array of countries or that utilize a methodology that can be modified to estimate productivity costs of specific subgroups of interest.

In this paper our aims are: 1) to present a transparent, generic model based on accepted analytic methods that allows users to assess the present value of lifetime earnings (PVLE) for select developed and emerging countries; 2) to discuss results from this model for a sample of diverse countries; and 3) to highlight an application of our model by incorporating mortality rates from the Benefits of Smoking Cessation on Outcomes Model (BENESCO) in order to estimate smoking-related lost productivity costs.

## Methods

We selected 29 countries to be included in the model based on their geographic diversity, and also to represent a range of developed and emerging countries. Countries were required to have a minimum amount of data available (i.e., at least life expectancy, economic participation, and wages) in order to be incorporated into the model; no data imputation was performed.

We used the following equations to estimate PVLE for paid work and household work:

(1) $$PVLE_{paid\;work\;\left(i,j\right)}=\sum_{i=s_{j}}^{n_{j}}\frac{\left(l_{i,j}*W_{i,j}\right)}{{\left(1+r\right)}^{i-s_{j}}}$$

(2) $$PVLE_{household\;work\;\left(i,j\right)}=\sum_{i=s_{j}}^{n_{j}}\frac{\left(\left(1-l_{i,j}\right)*h_{i,j}*w_{i,j}\right)}{{\left(1+r\right)}^{i-s_{j}}}$$

Where, j =, gender; sj =, the starting age for gender j; nj =, life expectancy for starting age for gender j; li,j =, economic activity rate for age i and gender j; Wi,j =, annual wages for age i and gender j; wi,j =, hourly wage for age i and gender j; hi,j =, hours of household work for age i and gender j; r =, discount rate.

Table 1 lists the 29 countries included and the types of data used for each country’s PVLE estimates.

**Table 1:** Countries included in model and data used for each country

**Region**^**1**^	**Country**	**Level of Development**^**2**^	**Life Expectancy**	**Labor Force Participation**	**Wages**			**CPI**	**Discount Rate**	**Can Include Household Labor**
					**Overall**	**By age**	**By sex**			
Africa	Egypt	Emerging	**x**	**x**			**x**	**x**	8.20 %	
	South Africa	Emerging	**x**	**x**	**x**			**x**	6.50 %	**x**
The Americas	Brazil	Emerging	**x**	**x**	**x**			**x**	4.80 %	
	Canada	Advanced	**x**	**x**		**x**	**x**	**x**	3.90 %	**x**
	Colombia	Emerging	**x**	**x**	**x**			**x**	8.60 %	
	Mexico	Emerging	**x**	**x**	**x**		**x**	**x**	6.20 %	**x**
	United States	Advanced	**x**	**x**		**x**	**x**	**x**	4.50 %	**x**
Asia	China	Emerging	**x**	**x**	**x**			**x**	14.30 %	**x**
	India	Emerging	**x**	**x**	**x**			**x**	11.70 %	**x**
	Indonesia	Emerging	**x**	**x**			**x**	**x**	7.10 %	**x**
	Israel	Advanced	**x**	**x**	**x**			**x**	3.00 %	
	Japan	Advanced	**x**	**x**			**x**	**x**	3.90 %	**x**
	Turkey	Emerging	**x**	**x**	**x**			**x**	7.80 %	**x**
Europe	Czech Republic	Advanced	**x**	**x**	**x**			**x**	8.90 %	
	Denmark	Advanced	**x**	**x**		**x**	**x**	**x**	4.90 %	
	Belgium	Advanced	**x**	**x**			**x**	**x**	5.00 %	**x**
	Finland	Advanced	**x**	**x**			**x**	**x**	7.90 %	**x**
	France	Advanced	**x**	**x**		**x**	**x**	**x**	3.30 %	**x**
	Germany	Advanced	**x**	**x**	**x**			**x**	4.60 %	**x**
	Netherlands	Advanced	**x**	**x**	**x**			**x**	4.50 %	
	Poland	Emerging	**x**	**x**	**x**			**x**	8.50 %	**x**
	Portugal	Advanced	**x**	**x**		**x**	**x**	**x**	2.40 %	**x**
	Russia	Emerging	**x**	**x**	**x**			**x**	10.00 %	
	Spain	Advanced	**x**	**x**		**x**	**x**	**x**	5.70 %	**x**
	Sweden	Advanced	**x**	**x**			**x**	**x**	6.40 %	**x**
	United Kingdom	Advanced	**x**	**x**		**x**	**x**	**x**	4.40 %	**x**
Oceania	Australia	Advanced	**x**	**x**			**x**	**x**	3.30 %	**x**
	New Zealand	Advanced	**x**	**x**			**x**	**x**	2.70 %	**x**
	Papua New Guinea	Emerging	**x**	**x**			**x**	**x**	4.30 %	

Sources:

(1) As defined by the United Nations Statistics Division, http://unstats.un.org/unsd/methods/m49/m49regin.htm#ftnb.

(2) As defined by the IMF, http://www.imf.org/external/pubs/ft/weo/2010/01/weodata/weoselagr.aspx#a001.

Notes:

Subject to data availability, the largest countries by population and level of development (advanced/emerging), in addition to further examples from each region, were included.

### Estimation of default model inputs

#### Life expectancy

The 2008 World Health Organization (WHO) Life Tables were used to obtain life expectancy values for all countries for ages 18 to 75 [17]. By design, the WHO life tables are stratified by sex and age, in 5-year increments. The WHO tables are generated based on a systematic review of all available evidence from surveys, censuses, sample registration systems, population laboratories and vital registration on levels and trends in mortality rates [17]. WHO applies a standard methodology to the analysis of all member state data. Because a person’s projected life expectancy depends on his or her current age, the PVLE model uses the expected average number of years of life left for a person at age i and gender j.

#### Economic activity rate

Labor force participation was derived from the economically active population, as presented by the International Labor Organization (ILO) [18]. For most countries, values from 2008 were used in our model; however, when data were not available, the next most recent year with complete data was selected. The labor force data, as reported by the ILO, were stratified by sex and age, in 5-year increments from ages 18 to 75. Our model assumed a labor force participation rate of 0 for all persons aged 76 years and older. The ILO refers to the “economically active population” as all persons of either sex who furnish the supply of labor for the production of goods and services during a specified time-reference period [18].

Categorization of groups such as the armed forces, members of religious orders, persons seeking their first job, seasonal workers or persons engaged in part-time economic activities may vary between countries. In certain countries, all or some of these groups are included among the economically active, while in others they are treated as inactive; by necessity, we followed these country-specific conventions. The data on economically active population generally exclude students, persons occupied solely in domestic duties in their own households, members of collective households, inmates of institutions, retired persons, persons living entirely on their own means, and persons wholly dependent upon others.

#### Wages

Wage data were gathered from a number of country-specific statistics websites. ILO estimates of country-specific wages were used when data from the corresponding national websites were not available. Wages were available for the overall population of each country, and several countries also had age- and sex-stratified data available. A country-specific Consumer Price Index (CPI) from the October 2009 International Monetary Fund (IMF) World Economic Outlook Database was used to update all wages to 2009 values. It was assumed that all labor force participants work full-time, as a common basis from which adjustments are possible.

#### Discount rates

The choice of appropriate discount rate in analyses of productivity costs is controversial and can be varied in this PVLE model. Social discount rates derived using a social rate of time preference methodology [19] were applied in the present value calculation. Discount rates for developing countries were considerably higher than those for developed countries; rates for emerging countries ranged from 4–14 % and rates for advanced countries ranged from 2–9 %.

#### Value of household work

Although not a primary focus of our calculations, for certain countries with available data, the model may also be modified to include the economic value of household work for persons not participating in the labor force. Time use surveys were used to provide national estimates of the number of hours spent on unpaid household work for each gender, when available [20-25]. The opportunity cost approach, i.e. estimating what a person’s labor would have been worth had those hours been spent in the paid labor market, was used to estimate the value, by gender, of time spent on household work. We also conducted sensitivity analyses that use the replacement cost, based on the average hourly wage for child care workers, personal care aides, and maids/housekeepers in the United States [26] rather than the market wage, to calculate PVLE for household labor using the 35–64 age group as an illustration.

#### Standardization of input data

Our model calculations account for each age from 18 to 75 years. Model inputs were not consistently reported across countries in uniform age groups or for each distinct age. We therefore assigned the best data available across age groups when needed (e.g., the life expectancy reported for the age group 20–24 was assigned to ages 20, 21, 22, 23, and 24). The model provides PVLE estimates, by sex and age, for 3 age groupings: 5-years, 10-years, or 3 broad categories (18–34, 35–64, and 65+ years), depending on the preference of the researcher using the model. For the PVLE calculations, the midpoint of each age range was used by our model. For the purposes of this paper, we report detailed model inputs and results using three large age groups for five countries as examples. Results are available for all countries and all 3 age groupings in an on-line Additional file 1: Appendix.

### Application of the model and BENESCO case study

**Figure 1:**
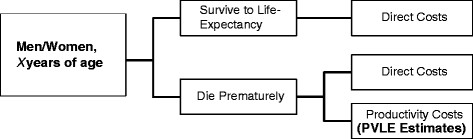
PVLE flow diagram.

Productivity costs due to premature mortality are a key inclusion in any economic evaluation from the societal perspective. Using age- and gender- specific mortality rates, productivity costs from our model can easily be incorporated (Figure 1). To illustrate the impact of incorporating productivity costs using our model, we estimate PVLE based on results from the BENESCO model. BENESCO is a Markov model that simulates the impact of a single quit attempt for different treatment options and estimates smoking-related morbidity, mortality, and direct costs [27]. BENESCO generates results for persons who have attempted to quit using various methods. For convenience, we therefore arbitrarily show results for smokers who attempt to quit without assistance (i.e., “cold turkey”), one of the selected strategies for smoking cessation included in BENESCO. We present the overall societal costs in two example countries (one developed and one emerging) broken down by direct and productivity costs. Sensitivity analysis of these results using the friction cost methodology was also conducted. A three-month friction period was assumed [9,10,28] and employee replacement costs, as reported by Wang and colleagues [29] included the costs of hiring and training a new employee ($8,819 in 2009 USD for the United States; adjusted to 3,659 Brazilian Reals for Brazil based on relative annual wages and average USD exchange rate in 2009).

## Results

Findings are presented for a sample of five countries drawn from developed and emerging countries, including Brazil, China, Egypt, the United Kingdom, and the United States. Selected model inputs for the five sample countries are presented in Table 2. For illustrative purposes, only sex-stratifications are shown. Not surprisingly, life expectancy is longer in developed countries, like the United Kingdom and in the United States, and is longer for women than for men; paid labor force participation is also consistently higher for men than it is for women. Annual paid wages are higher for developed than for emerging countries and, when stratification by sex was possible, are higher for men than for women.

**Table 2:** Select model inputs for 5 key countries

**Country**	**Life Expectancy- Men**^**a**^	**Life Expectancy- Women**^**a**^	**LF Participation- Men**^**b**^	**LF Participation- Women**^**b**^	**Annual Wages- Men**	**Annual Wages-Women**	**Currency**
Brazil	69.8	76.8	72.4 %	52.4 %	15,924	15,924	2009 Reals
China	72.1	75.7	79.7 %	67.5 %	29,213	29,213	2009 Yuan
Egypt	68.1	70.9	48.4 %	15.9 %	17,623	13,707	2009 Egyptian Pounds
United Kingdom	77.6	81.7	56.7 %	46.3 %	33,268	19,491	2009 British Pounds^c^
United States	75.7	80.7	72.3 %	58.9 %	42,224	34,164	2009 US Dollars^c^

[a] Life expectancy values used in the PVLE model vary by age person has lived until (in 5 year increments); these are for those <1 year old and are presented for illustrative purposes.

[b] Labor force participation values used in the PVLE model vary by age (in 5 year increments); these are overall figures for the total population and are presented for illustrative purposes. Values for Brazil and Egypt are for 2007; Values for China, the United Kingdom and the United States are for 2008.

[c] Wage inputs for the United Kingdom and the United States in the PVLE model vary by age (in 5 year increments); these are overall figures for the total working populations and presented here for illustrative purposes.

Table 3 presents PVLE estimates (excluding household labor costs) stratified by age (using three age groups) and sex for the five selected developed and emerging countries. PVLE estimates were highest for younger people partly because younger people had the most time ahead of them in the labor force and thus the most potential future productivity. PVLE estimates were higher for men than for women for primarily two reasons: (1) a higher percentage of men than women were typically in the work force; and (2) men typically were paid a higher wage than women. When the value of lost household labor is included in the model, using the United States as an example, PVLE estimates for the 35–64 year-old age group increase by approximately $120,000 for males and $194,000 for females. If the replacement cost of household labor method was used in our model instead of the opportunity cost approach, the PVLE estimates would have increased by $55,000 for males and $118,000 for females.

**Table 3:** Present value of lifetime future earnings estimates for select countries

**Country**	**Currency**	**Age Group**	**PVLE**	
			**Male**	**Female**
Brazil	2009 Brazilian Reals	18-34	273,400	200,901
		35-64	177,531	108,677
		65+	54,495	25,114
China	2009 Chinese Yuan	18-34	215,905	174,858
		35-64	174,806	94,237
		65+	31,756	7,223
Egypt	2009 Egyptian Pounds	18-34	211,527	49,328
		35-64	150,312	29,638
		65+	27,098	3,460
United Kingdom	2009 British Pounds	18-34	590,013	296,950
		35-64	366,281	161,067
		65+	37,707	10,085
United States	2009 US Dollars	18-34	757,640	510,682
		35-64	530,425	334,154
		65+	103,090	55,163

Note: Results are shown in 3 broad age categories, but the model allows for reporting in 5- and 10-year age groups as well. Estimates do not include household productivity costs.

**Figure 2:**
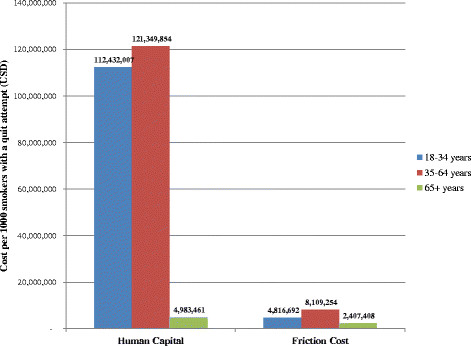
Lifetime productivity costs per 1000 smokers with a quit attempt by age, United States, human capital approach vs. friction cost approach.

**Figure 3:**
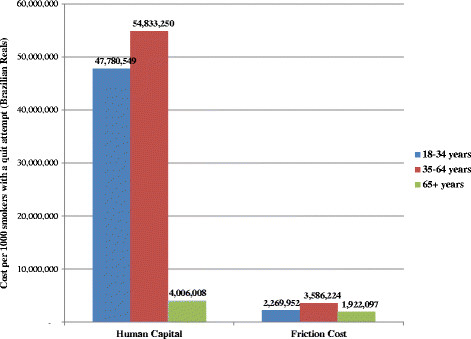
Lifetime productivity costs per 1000 smokers with a quit attempt by age, Brazil, human capital approach vs. friction cost approach.

Our model’s PVLE estimates were incorporated into the BENESCO model for two of these countries – the United States and Brazil – to estimate the total lifetime costs among smokers. The inclusion of productivity cost (excluding household labor) estimates from our model showed that productivity loss was a substantial share of the total cost burden of premature mortality due to smoking, with direct medical costs accounting for only 25 % of total lifetime costs in the United States and 33 % of total lifetime costs in Brazil. Figures 2 and 3 compare productivity cost results per 1,000 smokers undergoing a quit attempt for these two countries using human capital-based results from our model and using the friction cost method. These figures indicate that estimates using the human capital approach were substantially higher than those using the friction cost approach for the 18–34 and 35–64 age groups (23 and 15 times higher for the United States, and 21 and 15 times higher for Brazil, respectively). However, human capital productivity costs estimates for the 65+ age group were only approximately 2 times higher than friction costs for both countries.

## Discussion

In this paper, we present country-specific productivity cost estimates for several countries. Our human capital-based PVLE estimates for the US were slightly lower than those calculated by Grosse *et al*[15]. Their estimated PVLE (excluding household work) for males ranged from just under $16,000 to over $1,000,000 (2007 USD) depending on age; our estimates ranged from approximately $22,000 to $829,000 across similar age groups (age groupings not reported in this paper). This is likely due to our differing methodologies in estimating annual wages. Grosse *et al.* aggregated hourly wage estimates to an annual level based on estimated hours worked per week, whereas, to ensure a certain level of consistency across countries methodologically, we used average annual wages by age and sex. Estimates from our model for China were on par with the literature for the human capital-based method as well. For males between 35 and 64 for 5-year age groups, our model estimated PVLE to be between approximately 57,000 and 209,000 (2009 Chinese Yuan), while Sung *et al.* estimated a range of 27,350 and 264,000 (2003 Chinese Yuan) [30].

The most notable critique of the human capital approach is that it measures potential lost production instead of actual lost production, which could lead to significant overestimation of productivity costs due to premature mortality [9-11]. It has been argued that the friction cost methodology is more precise than the human capital methodology in that it captures actual productivity losses versus the total potential loss in the human capital approach [9,10]. However, the level of detail and extensive data required for this method (e.g., industry- or education-level data on average length of vacancy for an open position, disease-related average frequency and length of absence from work) made its application to our objective of estimating productivity costs across several countries unrealistic. We did include a sensitivity analysis using a rough approximation of the friction cost approach for the US and Brazil and found, as might be expected, substantially lower costs for lost productivity among those of working age. Further research into available sources of data to fully implement the friction cost approach would be valuable, such as length of the friction period for various occupations, availability of surplus labor, and whether positions are filled by unemployed workers or employed workers who are changing jobs (and thus generating a new friction period).

By its nature, our model is subject to certain limitations, namely surrounding data availability. It was not possible to stratify wages by age and sex in all countries, or to assess variation by occupation. Employment statistics did not distinguish between full-time and part-time work in most countries; our use of a full-time work assumption may overstate lost productivity costs. However, proportionate downward adjustments are possible to allow for data or assumptions about part-time work. Labor force participation rates and wages both vary based on general economic conditions, which would influence our estimates (e.g., higher estimates in good economic times, lower estimates in bad times). Due to this data variability, along with differences in epidemiology among diseases of interest and differences across health care systems, we recommend, in general, that researchers use the PVLE estimates to conduct single country analyses. Despite these known limitations, there has been recent interest in analysis of the transferability of economic evaluations across countries [31,32]. Thus, it may be possible to cautiously relate results between countries if special attention is given to assessing the comparability of model inputs and any key differences in country characteristics.

Our model evaluated lost productivity based on averages for general populations, including persons in the paid work force and those who are not, rather than only for those with specific diseases or comorbidities. Although these may limit the direct relevance of our model inputs and calculation to specific diseases, it strengthens the generalizability of our findings regarding the societal productivity costs. We also focused only on the value of lost productivity due to premature mortality. We recognize that indirect costs also should include morbidity costs, which were beyond the scope of our analysis. Finally, it could be argued that the actual cost to society from premature mortality would be production loss minus consumption loss, which would overstate productivity losses under the human capital approach.

We expanded on the traditional human capital model by making it possible to incorporate estimates of the value of household work, which has known limitations, including, for example, the transfer of gender wage differentials in the paid labor market to the production of household services [33]. Furthermore, we acknowledge that our use of an opportunity cost approach to estimate the value of household work rather than a replacement cost (i.e., the cost to hire someone to complete the household work) results in higher estimates of PVLE when household work is included, as exhibited by the sensitivity analysis that we conducted. As stated previously, due to data limitations we chose to use the opportunity cost approach in this model. As with any valuation based on age- and gender- specific wages, these PVLE estimates are biased against older and female populations. Despite the potential ethical considerations surrounding valuing one life more than another, it is still recommended to use age- and gender-specific wages where possible, as they are more targeted and accurate [5].

Also, in order to collect some input values from source documents written in other languages, we used Google Translate, which produced English versions that were imperfect and sometimes required additional interpretation.

Our analysis highlights the need for uniform data across countries. International organizations, such as the WHO and ILO, provide useful country-specific estimates for important model inputs such as life expectancy and labor force participation. However, where there are gaps in standardized data, country-specific data sources had to be used, which introduced some methodological variability into our results. Finally, the use of broader age categories leads to more imprecision in estimates of PVLE, so we recommend that researchers use the 5-year age brackets when feasible.

## Conclusions

Our results indicate important differences in human capital-based productivity costs by age and sex, and, as seen in our smoking case study, including productivity cost in a health economic evaluation can have a significant effect on the overall conclusions of level of burden. The inclusion of productivity cost estimates from our model in the BENESCO model of the burden of smoking showed that productivity loss due to premature mortality was a substantial share of the total cost burden of smoking in both the United States and Brazil, accounting for over 75 % and 67 % in each country, respectively.

Contributing to the existing productivity cost literature, our methods for estimating PVLE can be used across a wide range of emerging and developed countries using the well-accepted human capital approach. Currently, researchers can access productivity cost estimates for 29 countries available in an on-line Appendix (http://www.biomedcentral.com/bmcmedresmethodol/).

## Competing interests

This analysis was funded by Pfizer Inc., where Dr. Willke is employed and Dr. Marton, recently retired, was employed at the time this work was done. Dr. Menzin, Dr. Woodward, Mr. Menzin, and Ms. Federico are employees of Boston Health Economics Inc. who were paid consultants to Pfizer Inc. in connection with this analysis and the development of the manuscript.

## Authors’ contributions

JM, JPM, and RJW contributed to the conception and design of this study. JPM and RJW contributed to the provision of study materials (i.e., the BENESCO data). JM, JAM and VF contributed to the collection and/or assembly of data. JM, JAM, RMW and VF contributed to data analysis and interpretation. RMW and VF contributed to manuscript writing. JM, JPM, JAM and RJW contributed to manuscript revisions. All authors have given their final approval of the manuscript.

## Pre-publication history

The pre-publication history for this paper can be accessed here:

http://www.biomedcentral.com/1471-2288/12/87/prepub

## Supplementary Material

Additional file 1 Appendix. Click here for file
