# Supplementary material for: Lost productivity due to premature mortality in developed and emerging countries: an application to smoking cessation
Source: BMC Med Res Methodol. 2012 Jun 25;12:87. doi: 10.1186/1471-2288-12-87 (PMC3431987; doi:10.1186/1471-2288-12-87)
Supplement: Additional file 1 — Appendix. [file 1471-2288-12-87-S1.pdf]

Appendix: Present Value of Lifetime Earnings by Country

Australia

Present Value of Lifetime Earnings

Age Group	Male	Female
20-24	1,271,166	694,269
25-29	1,204,580	644,790
30-34	1,109,442	592,454
35-39	995,193	536,991
40-44	863,703	467,048
45-49	713,129	374,284
50-54	541,983	267,335
55-59	360,800	160,011
60-64	193,441	72,088
65-69	71,438	20,552
70-74	16,400	3,072
75+	4,302	806

Estimates do not include household productivity costs
Wages reported in 2009 Australian Dollars

Belgium

Present Value of Lifetime Earnings

Age Group	Male	Female
20-24	567,136	433,007
25-29	570,013	430,071
30-34	529,172	390,253
35-39	473,240	339,633
40-44	403,368	279,129
45-49	316,736	205,848
50-54	213,491	126,803
55-59	108,341	56,839
60-64	35,759	14,250
65-69	9,876	2,928
70-74	3,199	936
75+	550	227

Estimates do not include household productivity costs
Wages reported in 2009 Euros

Brazil

Present Value of Lifetime Earnings

Age Group	Male	Female
20-24	279,321	207,520
25-29	271,326	198,429
30-34	256,684	183,860
35-39	237,203	165,129
40-44	213,150	141,933
45-49	183,955	114,480
50-54	150,380	85,824
55-59	114,012	58,996
60-64	77,224	36,553
65-69	44,267	20,132
70-74	18,215	7,854
75+	3,376	1,322

Estimates do not include household productivity costs
Wages reported in 2009 Brazilian Real

Canada

Present Value of Lifetime Earnings

Age Group	Male	Female
20-24	960,465	547,279
25-29	1,024,527	575,003
30-34	975,543	539,721
35-39	911,311	496,875
40-44	833,259	442,711
45-49	715,472	365,926
50-54	535,884	261,502
55-59	340,652	152,791
60-64	159,788	60,044
65-69	52,733	12,725
70-74	25,687	6,198
75+	6,795	1,640

Estimates do not include household productivity costs

Wages reported in 2009 Canadian dollars

China

Present Value of Lifetime Earnings

Age Group	Male	Female
20-24	202,633	176,887
25-29	215,826	171,281
30-34	214,301	151,533
35-39	209,154	135,169
40-44	198,804	120,302
45-49	179,987	99,800
50-54	148,899	70,111
55-59	104,193	39,061
60-64	56,746	16,021
65-69	25,387	5,787
70-74	8,515	1,970
75+	1,198	292

Estimates do not include household productivity costs

Wages reported in 2009 Chinese Yuan Renminbi

Colombia

Present Value of Lifetime Earnings

Age Group	Male	Female
20-24	14,213,808	6,506,218
25-29	14,698,300	6,645,494
30-34	14,325,630	6,405,626
35-39	13,762,675	6,043,280
40-44	12,912,277	5,495,922
45-49	11,627,666	4,669,083
50-54	9,687,134	3,420,061
55-59	7,290,396	1,934,947
60-64	6,309,446	1,674,593
65-69	4,827,625	1,281,302
70-74	2,589,187	687,197
75+	729,461	193,607

Estimates do not include household productivity costs
Wages reported in 2009 Colombian pesos

Czech Republic

Present Value of Lifetime Earnings

Age Group	Male	Female
20-24	2,680,201	2,058,433
25-29	2,888,697	2,264,361
30-34	2,839,099	2,338,214
35-39	2,692,947	2,372,266
40-44	2,466,794	2,203,590
45-49	2,143,950	1,814,339
50-54	1,687,851	1,230,165
55-59	1,062,604	579,519
60-64	444,497	179,940
65-69	150,194	69,403
70-74	47,381	19,316
75+	6,030	1,573

Estimates do not include household productivity costs

Wages reported in 2009 Czech Republic Koruna

Denmark

Present Value of Lifetime Earnings

Age Group	Male	Female
20-24	7,532,475	5,491,911
25-29	7,346,172	5,333,564
30-34	6,947,437	5,019,524
35-39	6,365,814	4,567,745
40-44	5,626,295	3,980,768
45-49	4,722,542	3,251,821
50-54	3,630,795	2,346,261
55-59	2,305,354	1,310,745
60-64	1,100,898	480,384
65-69	472,264	189,398
70-74	140,066	47,128
75+	12,928	2,838

Estimates do not include household productivity costs

Wages reported in 2009 Danish Krones

Egypt

Present Value of Lifetime Earnings

Age Group	Male	Female
20-24	199,823	50,223
25-29	211,042	49,023
30-34	205,692	47,050
35-39	194,635	44,179
40-44	178,840	39,460
45-49	155,713	31,613
50-54	125,447	22,663
55-59	86,304	14,304
60-64	44,650	6,045
65-69	23,745	3,032
70-74	12,638	1,614
75+	3,542	452

Estimates do not include household productivity costs

Wages reported in 2009 Egyptian Pounds

Finland

Present Value of Lifetime Earnings

Age Group	Male	Female
20-24	441,924	320,463
25-29	446,952	325,690
30-34	431,780	322,656
35-39	404,518	312,272
40-44	364,633	289,032
45-49	312,994	247,538
50-54	242,646	188,046
55-59	156,138	112,871
60-64	73,164	43,106
65-69	25,486	7,650
70-74	9,315	2,196
75+	2,601	613

Estimates do not include household productivity costs

Wages reported in 2009 Euros

France

Present Value of Lifetime Earnings

Age Group	Male	Female
20-24	709,967	513,448
25-29	684,999	495,958
30-34	620,895	451,317
35-39	541,563	395,698
40-44	448,668	328,287
45-49	341,079	248,510
50-54	219,300	157,757
55-59	101,920	70,859
60-64	30,417	17,526
65-69	8,578	3,683
70-74	2,121	734
75+	178	59

Estimates do not include household productivity costs

Wages reported in 2009 Euros

Germany

Present Value of Lifetime Earnings

Age Group	Male	Female
20-24	749,279	489,744
25-29	739,217	475,962
30-34	700,304	449,281
35-39	637,822	412,974
40-44	557,755	360,539
45-49	459,442	290,449
50-54	341,640	205,338
55-59	206,460	112,586
60-64	83,592	38,503
65-69	21,980	8,753
70-74	7,143	2,486
75+	833	201

Estimates do not include household productivity costs

Wages reported in 2009 Euros

India

Present Value of Lifetime Earnings

Age Group	Male	Female
20-24	462,821	189,944
25-29	480,238	210,247
30-34	475,296	218,157
35-39	462,776	212,727
40-44	441,539	192,864
45-49	408,132	162,927
50-54	354,499	119,883
55-59	277,836	65,703
60-64	181,310	16,512
65-69	96,659	6,699
70-74	54,819	3,799
75+	16,056	1,113

Estimates do not include household productivity costs

Wages reported in 2009 Rupees

Indonesia

Present Value of Lifetime Earnings

Age Group	Male	Female
20-24	180,663,682	83,746,245
25-29	182,342,206	83,820,651
30-34	178,499,401	83,716,368
35-39	171,400,276	82,581,036
40-44	161,878,485	78,415,099
45-49	147,923,067	70,959,031
50-54	130,217,773	60,405,173
55-59	107,464,804	47,865,286
60-64	81,956,012	34,503,482
65-69	53,974,110	21,336,623
70-74	23,934,487	8,504,041
75+	4,932,769	1,395,253

Estimates do not include household productivity costs

Wages reported in 2009 Rupiah

Israel

Present Value of Lifetime Earnings

Age Group	Male	Female
20-24	1,898,562	1,606,249
25-29	1,906,853	1,569,960
30-34	1,778,203	1,438,984
35-39	1,619,349	1,283,505
40-44	1,419,505	1,097,379
45-49	1,192,485	886,667
50-54	936,824	650,565
55-59	654,203	400,533
60-64	374,897	186,152
65-69	151,343	52,603
70-74	37,793	9,633
75+	9,871	2,516

Estimates do not include household productivity costs
Wages reported in 2009 New Shekels

Japan

Present Value of Lifetime Earnings

Age Group	Male	Female
20-24	86,412,301	41,599,192
25-29	85,824,989	39,205,704
30-34	81,156,907	36,332,929
35-39	75,173,964	33,926,695
40-44	67,881,906	30,670,446
45-49	59,022,790	25,873,079
50-54	48,404,274	19,867,889
55-59	36,014,535	13,556,621
60-64	22,941,500	7,929,969
65-69	11,918,178	3,880,166
70-74	4,278,725	1,321,527
75+	591,777	151,593

Estimates do not include household productivity costs
Wages reported in 2009 Yen

Mexico

Present Value of Lifetime Earnings

Age Group	Male	Female
20-24	961,634	436,689
25-29	967,982	434,073
30-34	937,853	418,098
35-39	891,375	392,497
40-44	828,305	350,091
45-49	745,767	292,463
50-54	641,295	229,601
55-59	515,354	169,173
60-64	379,479	113,516
65-69	247,421	69,350
70-74	109,151	29,287
75+	19,672	4,558

Estimates do not include household productivity costs

Wages reported in 2009 Nuevo Pesos

Netherlands

Present Value of Lifetime Earnings

Age Group	Male	Female
20-24	810,081	519,393
25-29	783,314	490,285
30-34	730,204	448,310
35-39	661,024	398,330
40-44	576,337	338,621
45-49	474,635	265,969
50-54	353,124	183,084
55-59	214,646	98,911
60-64	93,717	35,389
65-69	33,221	9,071
70-74	10,833	2,442
75+	1,182	167

Estimates do not include household productivity costs
Wages reported in 2009 Euros

New Zealand

Present Value of Lifetime Earnings

Age Group	Male	Female
20-24	1,243,564	827,542
25-29	1,188,075	784,337
30-34	1,099,636	728,315
35-39	994,938	665,736
40-44	877,071	587,263
45-49	742,828	487,406
50-54	589,299	371,020
55-59	419,449	244,938
60-64	244,853	128,883
65-69	105,945	48,802
70-74	30,899	11,813
75+	2,414	673

Estimates do not include household productivity costs
Wages reported in 2009 New Zealand Dollars

Papua New Guinea

Present Value of Lifetime Earnings

Age Group	Male	Female
20-24	434,682	336,602
25-29	428,922	329,532
30-34	413,192	317,193
35-39	389,589	300,036
40-44	359,614	275,487
45-49	323,751	246,632
50-54	282,420	210,190
55-59	235,845	169,045
60-64	178,806	124,515
65-69	117,562	78,948
70-74	52,514	33,608
75+	12,199	6,666

Estimates do not include household productivity costs
Wages in 2009 Kina

Poland

Present Value of Lifetime Earnings

Age Group	Male	Female
20-24	385,444	319,814
25-29	404,985	340,013
30-34	387,752	328,846
35-39	357,487	303,988
40-44	313,568	259,731
45-49	256,877	196,028
50-54	187,669	117,785
55-59	112,198	51,635
60-64	53,258	23,282
65-69	23,903	11,053
70-74	8,733	3,636
75+	1,233	411

Estimates do not include household productivity costs
Wages reported in 2009 Zloty

Portugal

Present Value of Lifetime Earnings

Age Group	Male	Female
20-24	371,498	285,400
25-29	378,641	286,554
30-34	362,108	268,177
35-39	327,744	238,171
40-44	289,248	205,914
45-49	241,549	167,383
50-54	182,168	121,057
55-59	123,227	79,148
60-64	74,601	45,760
65-69	40,239	23,232
70-74	16,453	8,666
75+	3,094	1,355

Estimates do not include household productivity costs

Wages reported in 2009 Euros

Russia

Present Value of Lifetime Earnings

Age Group	Male	Female
20-24	2,167,946	1,919,708
25-29	2,269,820	2,060,956
30-34	2,229,695	2,070,892
35-39	2,138,349	2,008,152
40-44	1,971,583	1,828,969
45-49	1,678,651	1,473,223
50-54	1,294,494	1,003,069
55-59	814,764	522,396
60-64	353,398	205,963
65-69	132,808	69,355
70-74	73,100	38,174
75+	20,964	10,948

Estimates do not include household productivity costs
Wages reported in 2009 Roubles

South Africa

Present Value of Lifetime Earnings

Age Group	Male	Female
20-24	1,427,651	1,119,454
25-29	1,477,586	1,139,968
30-34	1,414,340	1,076,935
35-39	1,313,361	964,661
40-44	1,170,904	818,293
45-49	979,394	641,945
50-54	742,000	431,397
55-59	469,263	220,371
60-64	188,395	71,258
65-69	25,254	12,192
70-74	12,998	6,275
75+	3,563	1,720

Estimates do not include household productivity costs

Wages reported in 2009 Rands

Spain

Present Value of Lifetime Earnings

Age Group	Male	Female
20-24	331,631	206,137
25-29	359,359	216,251
30-34	363,739	205,599
35-39	347,997	186,506
40-44	316,601	160,623
45-49	269,631	126,673
50-54	204,258	86,278
55-59	121,332	45,022
60-64	45,144	14,974
65-69	8,748	2,599
70-74	1,700	539
75+	165	48

Estimates do not include household productivity costs
Wages reported in 2009 Euros

Sweden

Present Value of Lifetime Earnings

Age Group	Male	Female
20-24	565,204	429,695
25-29	569,322	435,428
30-34	550,036	422,890
35-39	516,138	397,282
40-44	469,418	359,800
45-49	408,372	309,681
50-54	330,749	244,244
55-59	232,201	162,115
60-64	120,650	73,861
65-69	39,306	15,864
70-74	10,818	3,027
75+	0	0

Estimates do not include household productivity costs

Wages reported in 2009 Krona

Turkey

Present Value of Lifetime Earnings

Age Group	Male	Female
20-24	199,532	71,910
25-29	202,667	67,787
30-34	191,579	62,424
35-39	173,649	55,549
40-44	148,100	46,356
45-49	116,921	37,201
50-54	86,974	28,472
55-59	61,331	20,451
60-64	39,846	12,828
65-69	23,338	6,942
70-74	12,326	3,666
75+	3,437	1,022

Estimates do not include household productivity costs
Wages reported in 2009 Turkish Lira

United Kingdom

Present Value of Lifetime Earnings

Age Group	Male	Female
20-24	569,634	299,594
25-29	593,391	295,060
30-34	588,181	279,243
35-39	541,878	250,725
40-44	478,200	215,231
45-49	386,768	171,338
50-54	283,410	118,469
55-59	175,117	63,649
60-64	78,864	22,415
65-69	28,428	7,376
70-74	8,373	1,824
75+	769	109

Estimates do not include household productivity costs

Wages reported in 2009 British pounds

United States

Present Value of Lifetime Earnings

Age Group	Male	Female
20-24	720,945	495,755
25-29	756,786	507,390
30-34	749,767	489,634
35-39	717,094	461,782
40-44	643,216	412,985
45-49	551,616	348,949
50-54	439,360	269,947
55-59	310,316	182,061
60-64	177,936	99,699
65-69	81,285	42,667
70-74	29,625	14,215
75+	4,278	1,620

Estimates do not include household productivity costs

Wages reported in 2009 US dollars
